# Impact of diet and exercise in growth restricted female rats on nephron endowment in male fetuses

**DOI:** 10.1113/EP092622

**Published:** 2025-10-25

**Authors:** Jessica F. Briffa, Sogand Gravina, Viktoria F. Richter, Dayana Mahizir, Kristina Anevska, Yeukai T. M. Mangwiro, James S. M. Cuffe, Fadi Charchar, Glenn D. Wadley, Deanne H. Hryciw, Karen M. Moritz, Mary E. Wlodek

**Affiliations:** ^1^ Department of Anatomy and Physiology The University of Melbourne Parkville VIC Australia; ^2^ Department of Microbiology, Anatomy, Physiology & Pharmacology LaTrobe University Bundoora VIC Australia; ^3^ School of Biomedical Sciences The University of Queensland St. Lucia QLD Australia; ^4^ Faculty of Science and Technology, School of Applied and Biomedical Sciences Federation University Australia Ballarat VIC Australia; ^5^ Institute for Physical Activity and Nutrition School of Exercise and Nutrition Sciences Deakin University Burwood VIC Australia; ^6^ Child Health Research Centre The University of Queensland South Brisbane QLD Australia

**Keywords:** exercise, growth restriction, high‐fat diet, kidney, nephron endowment, second generation

## Abstract

Uteroplacental insufficiency impairs kidney development and programs male‐onset cardiorenal disease, which can be transmitted to subsequent generations. Maternal lifestyle factors can independently influence fetal kidney development, highlighting that the lifestyle of growth‐restricted females can have further influence on F2 kidney development. In this study, we examined the impact of maternal growth restriction with or without additional lifestyle factors on F2 male kidney development. Uteroplacental insufficiency (Restricted) or sham (Control) surgery was performed on embryonic day 18 in Wistar–Kyoto rats. F1 females were fed a Chow or High‐fat diet from weaning (5 weeks of age). At 16 weeks, females were randomly allocated to an exercise group: no exercise (Sedentary); exercised prior to and during pregnancy (Exercise); or exercised only during pregnancy (PregEx). Females were mated at 20 weeks, with F2 male kidneys collected (embryonic day 20). Maternal growth restriction alone reduced nephron endowment by 29%, whereas additional lifestyle factors in Restricted dams reduced nephron endowment by ∼43%. Interestingly, Exercise only in High‐fat dams did not alter nephron endowment, which is likely to be attributable to significant kidney remodelling and/or enhanced resource availability. This study demonstrates that growth‐restricted dams that experience multiple maternal lifestyle factors (i.e. High‐fat diet and PregEx) impair the development of male F2 kidneys to a more severe extent than fetuses exposed to maternal growth restriction alone or a single maternal lifestyle factor in dams of normal birth weight (i.e. high‐fat feeding or exercise alone). Importantly, positive lifestyle interventions (Exercise) in a high‐fat environment compounded by adverse metabolic programming (Restriction) can be beneficial to fetal kidney development, which might prevent the transgenerational programming of cardiorenal disease.

## Abstract (highlights)

- **What is the central question of this study?**
  Uteroplacental insufficiency impairs kidney development, programming male‐onset cardiorenal disease, which can be transmitted transgenerationally. What impact do lifestyle factors in growth‐restricted dams have on second‐generation kidney development?
- **What is the main finding and its importance?**
  Lifestyle factors in growth‐restricted dams compromise fetal nephron endowment to a greater extent than growth restriction alone. Exercise prior to and throughout pregnancy maintains normal nephron endowment in high‐fat dams. Understanding the underlying mechanism(s) might offer therapeutic interventions for infants born small to improve their own and their children's cardiorenal health, breaking the transgenerational cycle.

## 1 INTRODUCTION

Epidemiological studies and animal models have well established that maternal perturbations during critical stages of development, such as uteroplacental insufficiency, occur at the expense of fetal organ development, resulting in a low birth weight and long‐term disease risk (Barker, 2007; Barker & Osmond, 1986; McMillen & Robinson, 2005). The fetal kidney is particularly susceptible to maternal perturbations (Dorey et al., 2014), with a low birth weight in humans and experimental rodent models being associated with a permanent reduction in nephron endowment, which predisposes them to an increased risk of developing cardiorenal disease in adulthood (Luyckx et al., 2013; Moritz et al., 2008; Wlodek et al., 2008). Kidney development, including nephrogenesis, is a complex but highly regulated process. Genes regulating renal branching morphogenesis, renal growth, cellular proliferation and apoptosis, in addition to epigenetic processes, are altered following several maternal perturbations in rodents (Abdel‐Hakeem et al., 2008; Cuffe et al., 2018; Doan et al., 2021, 2024; Gray et al., 2010; Shen et al., 2010; Singh, Cullen‐McEwen, et al., 2007; Singh, Moritz, et al., 2007; Welham et al., 2002), highlighting that multiple pathways contribute to the reduction in nephron endowment. However, very little is known about whether these gene changes are transmitted to the next generation, which can lead to the transgenerational transmission of F2 fetal nephron deficits (Gallo et al., 2013) and F2 male‐onset cardiorenal disease (Gallo et al., 2013; Master et al., 2014) in the rat.

Notably, individuals that were born small are at an increased predisposition to develop obesity, which can independently influence the health and development of their baby (Poston, 2012; Stothard et al., 2009). A key epidemiological study has demonstrated that maternal obesity reduces fetal kidney volume relative to body weight at late gestation (Luyckx & Brenner, 2010), which suggests that nephron endowment might be impaired and could lead to renal dysfunction (Hsu et al., 2014). Animal studies support the idea of a connection between maternal obesity and offspring renal function. Specifically, maternal obesity in rats programs glomerulosclerosis and enhances albuminuria in adult offspring (Jackson et al., 2012). A recent study reports that although maternal obesity does not impact fetal nephron endowment in rats, it does increase markers of renal stress, inflammation and apoptosis (Zhou et al., 2021). Thus, although there is evidence that maternal obesity can impact fetal kidney development, additional studies are required to elucidate the full impact of maternal obesity on the developing fetal kidney. Furthermore, the additive effect of maternal obesity in mothers who were born growth restricted remains relatively unknown.

Given that obesity can independently impact offspring renal function and that growth‐restricted mothers are more likely to become obese, this collectively highlights the need for relatively simple lifestyle interventions in mothers at risk to improve the health and development of their child. Moderate‐intensity exercise in pregnant women for ≥30 min day^−1^ reduces the risk of pre‐eclampsia, glucose intolerance and gestational diabetes mellitus (American College of Obstetricians and Gynecologists, 2003; Brankston et al., 2004; Saftlas et al., 2004). Likewise, a study in rats has demonstrated the benefits of maternal exercise on improving offspring insulin sensitivity (Carter et al., 2013). However, the effects of maternal exercise to negate the impact of maternal growth restriction and/or maternal obesity on fetal nephron endowment is unknown.

We have previously demonstrated that F1 growth‐restricted mothers transmit cardiorenal dysfunction to F2 male, but not female, offspring (Gallo et al., 2013; Master et al., 2014). This sex‐specific programming of disease is a common phenomenon in the field, which might be attributable to an in utero adaptation to maintain normal organ growth, development and function in female fetuses. However, it remains unknown whether maternal lifestyle factors (i.e. obesity and/or exercise) can also influence this F2 male phenotype. Therefore, in this study we examined the impact of maternal growth restriction, high‐fat feeding and exercise (individually or in combination) on F2 male fetal kidney development.

## 2 MATERIALS AND METHODS

### 2.1 Ethical approval

All experiments were approved by the University of Melbourne animal ethics sub‐committee (AEC: 1212639) before commencement, following the National Health and Medical Research Council (NHMRC) Australian code for the care and use of animals for scientific purposes. This study was performed in accordance with the policies of *Experimental Physiology* regarding animal experimentation and complies with their animal ethics checklist. Every effort was made to minimize the pain and distress of the animals.

### 2.2 Animals

We used our well‐established surgical model of late‐gestation intrauterine growth restriction in Wistar–Kyoto rats (Anevska et al., 2015, 2019; Briffa et al., 2017; Cuffe et al., 2018; Doan et al., 2024; Gallo et al., 2013, 2012; Mahizir et al., 2021, 2020; Master et al., 2014; Mazzuca et al., 2010; Moritz et al., 2009; O'Dowd, Wlodek, et al., 2008; Wadley et al., 2016; Wlodek et al., 2007, 2005). Wistar–Kyoto rats (8 weeks of age) were obtained from the Biological Research Facility at the University of Melbourne, housed in an environmentally controlled room (19°C–22°C) under a 12 h–12 h light–dark cycle (lights on at 07.00 h) and provided with standard food pellets and tap water ad libitum. Female rats were mated overnight with breeder males, and the presence of sperm in the vaginal smear the following morning indicated successful mating and was taken as day 1 of gestation.

On day 18 of gestation (E18; term = 22 days), pregnant rats underwent bilateral uterine vessel ligation surgery, as described previously (Wlodek et al., 2005). In brief, F0 females were anaesthetized with 4% isoflurane (Baxter Healthcare; Old Toongabbie, NSW, Australia) and 650 mL min^−1^ oxygen flow (reduced to 3.2% isoflurane and 250 mL min^−1^ oxygen flow when suturing, to aid in the recovery of the animal). Once the appropriate depth of anaesthesia was achieved (determined by lack of withdrawal following pedal pinch), uteroplacental insufficiency (bilateral uterine vessel ligation; offspring termed ‘Restricted’) or sham (offspring termed ‘Control’) surgery was performed. To provide pain relief postoperatively, Marcaine (0.125% bupivacaine; AstraZeneca, North Murarrie, QLD, Australia) was administered by local infiltration along the edges of the skin and muscle prior to closure. Immediately following surgery, the animals were constantly observed until they regained the righting reflex and were then monitored hourly on the day of surgery, then twice daily for 3 days postoperatively.

Dams were allowed to deliver naturally at term. Female body weights were recorded as a litter average at birth (PN1), and individual weights were recorded (on PN7, PN14 and PN35) after toe clipping (on PN7; approved procedure on AEC: 1212639) for identification; toe clipping was chosen in order for animals to be identified from PN7 for individual growth trajectories, whereas other methods can only be used to identify older animals (i.e. ear notching after PN14).

After allowing the F1 Restricted females to wean naturally (postnatal day 35; O'Dowd, Kent, et al., 2008; Wlodek et al., 2007), littermate first generation (F1) Control and Restricted females were allocated randomly to one of two diets (Chow or High‐fat; from Specialty Feeds, Glen Forest, WA, Australia) that were matched for protein content. Specifically, rats allocated to the Chow diet were provided with ad libitum access to AIN93G (7% fat, 19.4% protein, 56.9% carbohydrate with 16.1 MJ kg^−1^ digestible energy; 16% digestible energy from lipids) for the study duration. Rats allocated to the High‐fat diet were provided with ad libitum access to a mixture of two Semi‐purified High‐fat diets for the study duration [SF03‐020 (23% fat, 19.4% protein, 56.9% carbohydrate with 19.5 MJ kg^−1^ digestible energy; 43% digestible energy from lipids) and SF01‐028 (22.6% total fat, 19% protein, 56.9% total carbohydrates with 19.9 MJ kg^−1^ digestible energy; 43% digestible energy from lipids)].

**FIGURE 1:**
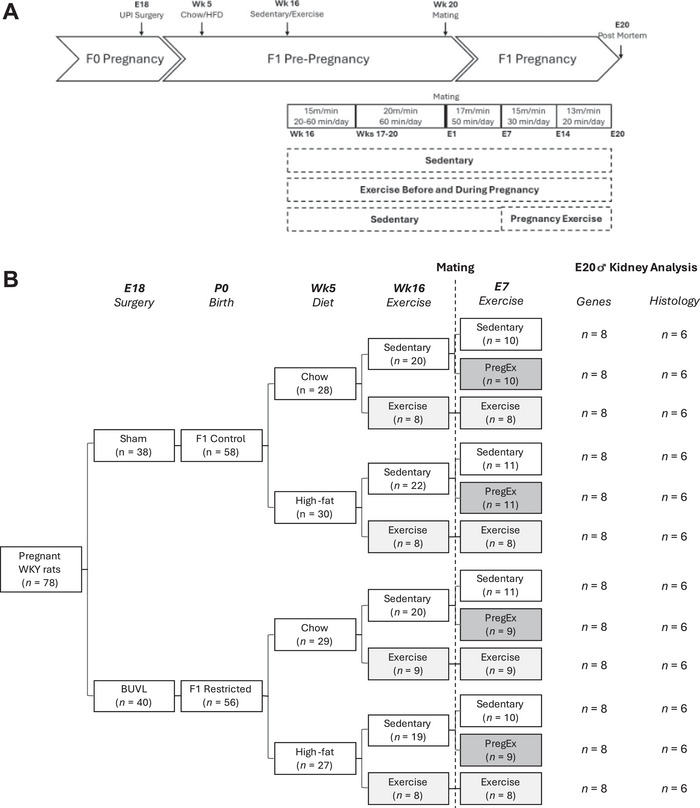
Study design. Flow chart (a) of the experimental protocol, indicating the allocation of F1 rats between the different treatments, diets, and exercise groups (b). Abbreviations: BUVL, bilateral uterine vessel ligation; E, embryonic day; HFD, high‐fat diet; P, postnatal day; UPI, uteroplacental insufficiency; Wk, week.

At 16 weeks of age, F1 females were further allocated randomly to one of three exercise regimes: no exercise (Sedentary); exercise prior to and throughout pregnancy (Exercise); or exercise only during pregnancy (PregEx; sedentary prior to and in the first week of pregnancy, then exercised from E7 to E19). At 20 weeks of age, F1 females were mated with breeder males that were not used to generate F0 pregnancies. Study design and sample size per group are shown in Figure 1. All animals were generated concurrently.

### 2.3 Exercise training

For the duration of their exercise regime, F1 females exercised on a motorized treadmill (Columbus Instruments, Columbus, OH, USA) for 5 days each week followed by 2 days of rest. This exercise regimen closely represents women performing moderate‐intensity aerobic exercise on 4 or 5 days a week, which is consistent with the American College of Obstetricians and Gynaecologists recommendations (Artal & O'Toole, 2003). On the first training day, rats allocated to the Exercise group ran for 20 min at 15 m min^−1^, with an additional 10 min applied on each subsequent day until day 5 of week 1. From day 1 of week 2 until mating, the rats exercised for 60 min day^−1^ at 20 m min^−1^. After mating, rats exercised for 50 min day^−1^ at 17 m min^−1^ for week 1 of pregnancy, 30 min day^−1^ at 13 m min^−1^ for week 2 of pregnancy, and 20 min day^−1^ at 11 m min^−1^ for week 3 of pregnancy. Females allocated to the PregEx group remained sedentary prior to mating and for the first week of pregnancy, then underwent exercise in weeks 2 and 3 of pregnancy at the same intensities and durations as the Exercise group. Rats were encouraged to run by blowing compressed air near the base of their tail (Anevska et al., 2019; Mahizir et al., 2021, 2020; Mangwiro, Briffa et al., 2018, 2021; Mangwiro, Cuffe et al., 2018; Mangwiro et al., 2019). Sedentary rats were placed on a stationary treadmill for the same duration as the exercising rats. We have previously published the maternal metabolic (Mahizir et al., 2020), cardiorenal (Mahizir et al., 2021) and bone (Anevska et al., 2019) phenotypes and the placental (Mangwiro, Briffa et al., 2018, 2021; Mangwiro, Cuffe et al., 2018; Mangwiro et al., 2019) outcomes of these exercise regimes.

### 2.4 Post‐mortem examination

On E20, F1 females were anaesthetized with an overdose of ketamine (100 mg kg^−1^; Parnell Laboratories, Alexandria, NSW, Australia) and xylazine (30 mg kg^−1^; Troy Laboratories, Smithfield, NSW, Australia) and their uterus was exposed. F2 fetuses were weighed and killed by decapitation, with fetal sex verified by sex‐determining region Y (*Sry*) qPCR (Mangwiro, Cuffe et al., 2018). Kidneys from F2 males were excised and weighed, with the left kidney frozen immediately in liquid nitrogen, then stored at −80°C, and the right kidney fixed in 10% neutral buffered formalin. For analyses, one male from each litter whose weight was closest to the male litter average was chosen, with each sample representing a single animal per litter (i.e. *n* = 1 per litter). The dam was then killed by cardiac puncture, and the dorsal fat pads and gastrocnemius muscles were excised and weighed. Tibial length was recorded to assess adipose and gastrocnemius mass‐to‐body length ratio. Only F2 males were investigated in the present study because we have previously shown that F2 males go on to develop cardiorenal disease in adulthood, from which the F2 females are protected (Gallo et al., 2013; Master et al., 2014).

### 2.5 Maternal plasma analysis

Maternal plasma leptin concentration was determined using ELISA (Signosis, Santa Carla, CA, USA) as described previously (Mahizir et al., 2020), with a minimum sensitivity of 8 pg ml^−1^. For analysis, seven or eight dams per group were analysed for plasma leptin concentrations (*n* = 8 for all, except Chow Control Exercise, Chow Restricted PregEx and High‐fat Restricted PregEx, for which *n* = 7).

### 2.6 Renal stereology

Fixed kidneys were processed into paraffin blocks, then sectioned sagittally to exhaustion at 5 µm thickness. The number of glomeruli was then estimated by sampling every 10th and 11th section using the physical dissector/fractionator method, as described previously (Akison et al., 2020). To remove sample bias, the paraffin blocks were code‐blinded and only decoded after all samples were counted. Prior to analysis, power calculations were performed using published data on late‐stage embryos by us (Dickinson et al., 2007; Gallo et al., 2013) and others (Wang et al., 2023). To achieve 80% power at a significance of α = 0.05, *n* = 5 was determined to be adequate to identify differences between groups. For analysis, five or six males per group were analysed for the number of nephrons (*n* = 6 for all, except High‐fat Control Exercise, for which *n* = 5), with *n* = 1 representing one pup from one litter.

### 2.7 Kidney gene abundance

Total RNA and DNA were extracted from frozen kidneys using commercially available phenol‐free Isolate II RNA/DNA/Protein kit (Bioline; Alexandria, NSW, Australia). RNA was reverse transcribed into complementary DNA using kits by Applied Biosystems (Scoresby, VIC, Australia). Real‐time PCR was used to determine the relative abundance of known nephrogenesis genes that regulate angiogenesis [*Vegfa*, *Kdr*, *Flt1* and *Hif1a* (Schley et al., 2015; Tufro et al., 1999)], renal growth [*Tgfb1*, *Wnt11* and *Wnt4* (Oxburgh et al., 2004)], branching morphogenesis [*Gdnf* and *Ret* (Costantini, 2010)] and apoptosis and proliferation [*Bax*, *Bcl2*, *Casp3*, *Tp53* and *Ki67* (Carev et al., 2006)] using TaqMan Gene Expression Assays probes (Life Technologies; Mulgrave, VIC, Australia). Given that we have demonstrated previously that maternal growth restriction, exercise and diet independently alter fetal steroidogenesis (Mangwiro et al., 2021), we also investigated changes in renal stress‐responsive genes (*Scnn1a*, *Sgk1*, *Hsp90aa1*, *Hsd11b2*, *Nr3c1* and *Nr3c2*) that are known to regulate kidney development and/or function (Celi et al., 2012; Cuffe et al., 2016; Pearce, 2003) using TaqMan Gene Expression Assays probes (Life Technologies; Mulgrave, VIC, Australia). Telomere genes of interest (*Tert* and *Terc*) were purchased from Life Technologies using previously designed probes (Booth et al., 2018). Real‐time PCR data were normalized to the geometric mean of two housekeeping genes using commercially available probes (Life Technologies): Glyceraldehyde 3‐phosphate dehydrogenase (*Gapdh*) and β‐actin (*Actb*). HotStart DNA Taq Polymerase was activated by heating samples to 95°C for 10 min, followed by 40 cycles of 95°C for 15 s and 60°C for 60 s. Relative changes in mRNA abundance were quantified using the 2^−ΔΔ^ *^Ct^* method and reported in arbitrary units normalized to Chow Sedentary Control male values. *Gapdh* and *Actb* were not different between Treatments, Diets or Exercises. For analysis, eight males per group were analysed for gene expression, with *n* = 1 representing one pup from one litter.

### 2.8 Telomere length

Telomere length was determined in extracted DNA by the telomere (T) to single‐copy gene (S) T/S ratio method (Booth et al., 2018) using SYBR green (Bioline). Briefly, the square of the cycle threshold (*Ct* ^2^) for the single‐copy gene *36b4* (‘S’) that is present only once in the genome is divided by the *Ct* ^2^ of *Tel1* (‘T’), which reflects telomere length (Cawthon, 2002). This yields the T/S ratio, a measure of average telomere length. DNA polymerase was activated by heating to 95°C for 5 min, followed by 40 cycles of 95°C for 10 s and 60°C for 50 s. For analysis, eight males per group were analysed for gene expression, with *n* = 1 representing one pup from one litter.

### 2.9 Statistical analysis

Given the complexity of the study, we initially sought advice from the School of Mathematics and Statistics at the University of Melbourne, who agreed with the statistical approach taken to answer our specific research questions. Normality testing was performed prior to statistical analysis using the Shapiro–Wilk test, and significant outliers were identified and removed following Grubbs’ test if they were >3SD from the mean. If the data failed normality testing, the data were transformed by either the square root or log_10_ and reanalysed. Statistical analysis was performed using SPSS Statistics 22 (IBM; St Leonards, NSW, Australia), GraphPad (Boston, MA, USA) or Excel (Microsoft; North Ryde, NSW, Australia) where appropriate. All data are presented as the mean ± SD, and statistical significance was set at *p *< 0.05. Given the small sample size for analyses (*n* = 5–8) and the large number of experimental groups (12) in this study, it is important to acknowledge there is potential for type II errors.

#### 2.9.1 Maternal outcomes

F1 female average litter birth weight was assessed using an unpaired *t*‐test.

Postnatal body weights failed normality testing, even after transformations. Given that there is no non‐parametric test equivalent to a mixed‐model two‐way ANOVA, we adjusted the significant *p*‐value for this dataset to *p *< 0.01. To assess differences in F1 body weight to weaning (PN35), a two‐way ANOVA was performed to determine differences between treatment and age. If a main Treatment effect was present, the two‐way ANOVA provided the *p*‐value. If a main Age effect was present, a one‐way ANOVA with Tukey's *post hoc* test was used to identify differences across postnatal ages. If an interaction was observed, the data were split further to identify Treatment effects within each Age using an unpaired *t*‐test, and a one‐way ANOVA with Tukey's *post hoc* test was used to determine Age effects in Control and Restricted groups.

Fifteen‐week body weight failed normality testing, even after transformations. Given that there is no non‐parametric test equivalent to a two‐way ANOVA, we adjusted the significant *p*‐value for this dataset to *p *< 0.01. To examine whether diet altered F1 weight at 15 weeks, a two‐way ANOVA was performed to determine differences between Treatment and Diet. If a main Treatment or Diet effect was present, the two‐way ANOVA provided the *p*‐value.

To identify differences in mating weight, a two‐way ANOVA was performed between Treatment and Exercise within each Diet. If a main Treatment or Exercise effect was present, the two‐way ANOVA provided the *p*‐value. To determine any differences between Diets, the data were split by Exercise, and a two‐way ANOVA was conducted to report main Diet effects within each exercise regime. If an interaction was observed, the data were split to identify Diet effects within each Exercise regime and Treatment using an unpaired *t*‐test.

For post‐mortem data, we initially performed a two‐way ANOVA to identify differences between Treatment and Exercise within each Diet. If a main Treatment effect was present, the two‐way ANOVA provided the *p*‐value. If a main Exercise effect was present, a one‐way ANOVA with Tukey's *post hoc* test was used to identify Exercise differences. If an interaction was observed, the data were split further to identify Treatment effects within each Exercise group using an unpaired *t*‐test, and a one‐way ANOVA with Tukey's *post hoc* test was used to determine Exercise effects in Control and Restricted groups. Given that we were only interested in Exercise and/or PregEx differences compared with the Sedentary group, we only draw reference to those in text. To determine any differences between Diets, the data were split by Exercise, and a two‐way ANOVA with Tukey's *post hoc* test was conducted to report main Diet effects within each exercise regime. If an interaction was observed, the data were split to identify Diet effects within each Exercise regime and Treatment using an unpaired *t*‐test. If a dataset failed the Shapiro–Wilk test after square root or log_10_ transformations, the significant *p*‐value was adjusted to *p *< 0.01 for a two‐way ANOVA because there is no comparable non‐parametric test; this was done for gastrocnemius weight and maternal plasma leptin.

#### 2.9.2 Fetal outcomes

Given that we were interested specifically in how the experimental groups differ from our control group (i.e. Chow Sedentary Control) for F2 male fetal kidney outcomes and not whether there were any between‐experimental‐group effects, we performed a one‐way ANOVA with Dunnett's *post hoc* test. If a dataset failed the Shapiro–Wilk test after square root or log_10_ transformations, the data were analysed with a Kruskal–Wallis test with Dunn's *post hoc* test.

Correlations between gene expression, telomere length and nephron number were determined using Spearman's non‐parametric correlation coefficient (no assumptions regarding data distribution). Initially, all groups were combined to investigate whether there is a relationship between expression of different pairs of genes and the number of nephrons, regardless of maternal growth restriction or maternal lifestyle factors. The relationships within each group (i.e. Control and Restricted), Diet (i.e. Chow and High‐fat) and Exercise (i.e. Sedentary and Exercise and PregEx) were then examined separately to explore whether a certain correlation is present in one group and is absent/altered in the other group, potentially indicating disruption attributable to maternal growth restriction, high‐fat feeding and/or exercise. Correlations were denoted as no (0.0 ≤ 0.1), weak (≥0.1 ≤ 0.3), moderate, (≥0.3 ≤ 0.50), strong (≥0.5 ≤ 0.8) or very strong (≥0.8–1.0) correlations.

## 3 RESULTS

### 3.1 Maternal parameters

**FIGURE 2:**
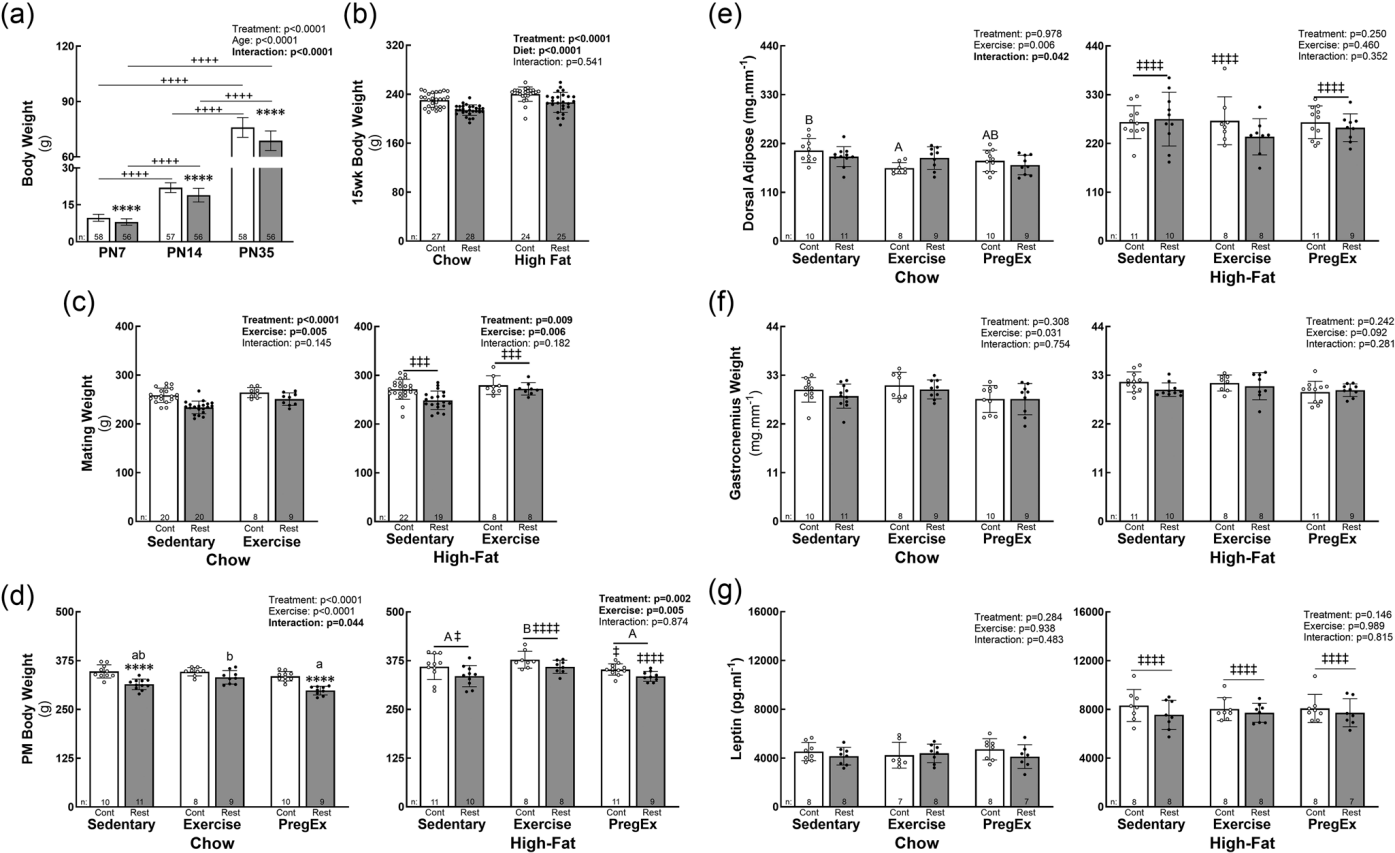
Maternal body weight and adiposity. (a) F1 Control (Cont) and Restricted (Rest) female postnatal (PN) body weights through to weaning (58 and 56 females, respectively; raw data included in Table S2). Data were analysed with a mixed‐effects two‐way ANOVA. (b, c) Body weights at 15 weeks of age following dietary intervention at PN35 (b; Chow Control = 27, Chow Restricted = 28, High‐fat Control = 24, High‐fat Restricted = 25) and at mating following exercise intervention (c; Chow Sedentary Control = 20, Chow Sedentary Restricted = 20, Chow Exercise Control = 8, Chow Exercise Restricted = 9, High‐fat Sedentary Control = 22, High‐fat Sedentary Restricted = 19, High‐fat Exercise Control = 8, High‐fat Exercise Restricted = 8). (d–f) Maternal body weight (d), in addition to relative dorsal fat (e) and gastrocnemius (f) weights at post‐mortem examination (PM) following exercise during pregnancy intervention (Chow Sedentary Control = 10, Chow Sedentary Restricted = 11, Chow Exercise Control = 8, Chow Exercise Restricted = 9, Chow PregEx Control = 10, Chow PregEx Restricted = 9, High‐fat Sedentary Control = 11, High‐fat Sedentary Restricted = 10, High‐fat Exercise Control = 8, High‐fat Exercise Restricted = 8, High‐fat PregEx Control = 11, High‐fat PregEx Restricted = 9). (g) Maternal leptin concentrations at embryonic day (E)20 (Chow Sedentary Control = 8, Chow Sedentary Restricted = 8, Chow Exercise Control = 7, Chow Exercise Restricted = 8, Chow PregEx Control = 8, Chow PregEx Restricted = 7, High‐fat Sedentary Control = 8, High‐fat Sedentary Restricted = 8, High‐fat Exercise Control = 8, High‐fat Exercise Restricted = 8, High‐fat PregEx Control = 8, High‐fat PregEx Restricted = 7). Data were analysed by a series of two‐way ANOVAs to identify the differences between Treatment (maternal birth weight) and Exercise (split by Diet) and between Treatment (maternal birth weight) and Diet (split by Exercise). Data presented as the mean ± SD. Differences between Control and Restricted are denoted with an asterisk (*), differences between postnatal ages is denoted with a plus (+), differences between Chow and High‐fat are denoted by a double dagger (‡), and differences across exercises are denoted by different letters, where ‘A/a’ is different to ‘B/b’ but not to ‘AB/ab’. */+/‡*p *< 0.05, **/++/‡‡*p *< 0.01, ***/+++/‡‡‡*p *< 0.001 and ****/++++/‡‡‡‡ *p *< 0.0001. Detailed information [comparison group sample sizes and means, mean difference, 95% confidence interval and *p*‐values (where appropriate)] regarding significant differences can be found in the Statistical Summary Document.

Consistent with our previous studies, F1 Restricted females were lighter at birth [−15.9%; Control = 4.18 ± 0.28 g (*n* = 38 litters) and Restricted = 3.52 ± 0.25 g (*n* = 40 litters), *p *< 0.0001; raw data included in Table S1] and remained lighter than Controls through to weaning (−9.5% to 17.5%; Figure 2a; raw data included Table S2). Regardless of their diet and/or exercise allocations, F1 Restricted females remained lighter at 15 weeks of age [−6.3%; 95% confidence interval (CI) (−19.72 g, −10.07 g); Figure 2b], mating [−6.4%; 95% CI for Chow (−26.82 g, −11.39 g) and High‐fat (−26.36 g, −3.89 g); Figure 2c] and post‐mortem [−7.3%; 95% CI for Chow Sedentary (−51.18 g, −15.22 g), Chow PregEx (−55.73 g, −17.92 g) and High‐fat (−32.19 g, −7.82 g); Figure 2d], apart from Chow Exercise Restricted dams, which were not different from Control counterparts. The timing of catch‐up growth in growth‐restricted offspring is known to program their health independently (Barker et al., 2005), highlighting that our observation of no ‘catch‐up’ growth suggests no beneficial and/or negative impacts of postnatal growth on maternal health outcomes.

Consumption of a high‐fat diet from PN35 increased body weight by 15 weeks of age [+5.1%; 95% CI (6.43 g, 16.08 g); Figure 2b], which remained higher than Chow counterparts at mating [+6.4%; 95% CI for Sedentary (6.18 g, 21.39 g) and Exercise (8.50 g, 28.64 g); Figure 2c]. At post‐mortem examination, body weight remained greater than Chow counterparts [+7.3%; 95% CI for Sedentary (1.68 g, 31.72 g), Exercise (16.63 g, 40.97 g), PregEx Control (2.04 g, 32.32 g) and PregEx Restricted (20.07 g, 52.73 g); Figure 2d], which coincided with increased adiposity [+46.7%; 95% CI for Sedentary (49.72, 98.88 g), Exercise Control (57.15 g, 156.3 g) and PregEx (−66.06 g, 104.6 g); Figure 2e] and plasma leptin concentrations [+81.4%; 95% CI for Sedentary (−1318 pg mL^−1^, 174.4 pg mL^−1^), Exercise (2909 pg ml^−1^, 4220 pg mL^−1^) and PregEx (−1260 pg mL^−1^, 306.9 pg mL^−1^); Figure 2g], irrespective of maternal exercise and birth weight.

F1 females that underwent Exercise from 15 weeks of age were heavier than Sedentary counterparts by mating [+5.4%; 95% CI for Chow (3.54 g, 18.98 g) and High‐fat (4.81 g, 27.28 g); Figure 2d]. However, post‐mortem body weight was only increased in High‐fat Exercise dams compared with High‐fat Sedentary dams [+6%; 95% CI (2.48 g, 38.88 g); Figure 2d], irrespective of birth weight, which did not correspond to alterations in adiposity (Figure 2e), skeletal muscle mass (Figure 2f) or plasma leptin concentrations (Figure 2g), suggesting that the increased body weight in High‐fat Exercise dams is not attributable to increased lean muscle mass and is likely to be attributable a separate mechanism, such as differences in water weight, glycogen storage, muscle inflammation and/or blood volume (Mora‐Rodriguez et al., 2016), which was not examined in the present study. Adiposity was reduced in Chow Exercise Control dams [−19.4%; 95% CI (−71.8 g, −7.18 g); Figure 2e]; however, this did not coincide with alterations in body weight or changes in skeletal muscle mass, highlighting the benefit of exercise on reducing adiposity in healthy women of normal birth weight.

### 3.2 Kidney weight and number of nephrons

**FIGURE 3:**
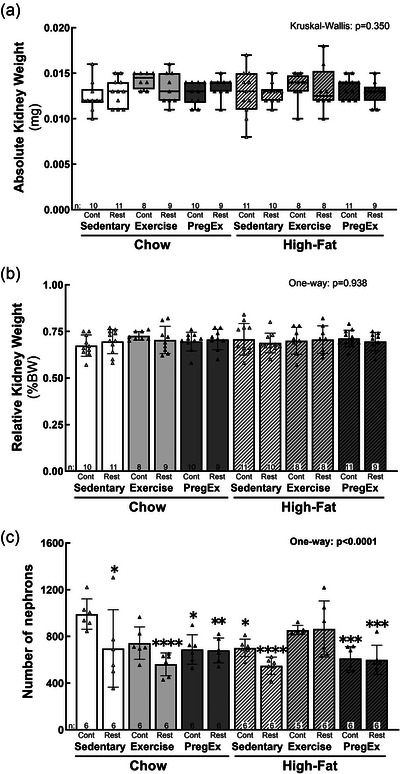
Kidney weight and nephron endowment. Absolute (a) and relative (b) F2 male fetal kidney weight (*n* = 8–12 litters in each group) and the number of nephrons (c) (*n* = 6 per group except for High‐fat Exercise Control, where *n* = 5, with *n* = 1 representing one pup from one litter) whose mothers were Control (Cont) or Restricted (Rest). Data were analysed with a one‐way ANOVA with Dunnett's *post hoc* test (normally distributed) or the Kruskal–Wallis test with Dunn's *post hoc* test (not normally distributed) to identify differences between experimental groups compared with control (Chow Sedentary Control). Data are presented as the mean ± SD or median (interquartile range). Significant differences between Chow Sedentary Control and experimental groups are indicated as follows: *p < 0.05, **p < 0.01, ***p < 0.001 and ****p < 0.0001. Detailed information [comparison group sample sizes and means, mean difference, 95% confidence interval and *p*‐values (where appropriate)] regarding significant differences can be found in the Statistical Summary Document.

We have previously demonstrated that Exercise in Chow dams increases fetal weight, with no effects of maternal growth restriction or high‐fat feeding (Mangwiro, Cuffe et al., 2018). Absolute (Figure 3a) and relative (Figure 3b) kidney weights were not different between experimental groups compared with Chow Sedentary Control males. The number of nephrons was reduced by 29.7% in Chow Sedentary Restricted animals [*p *= 0.014; 95% CI (−546.1, −43.22); Figure 3c] compared with Chow Sedentary Control males, which is consistent with our previous study (Gallo et al., 2013). A similar degree of reduced nephron endowment was also observed in F2 kidneys of normal birth weight mothers exposed to Exercise [Chow Exercise Control; −25.1%, *p *= 0.053; 95% CI (−500.6, 2.3)], PregEx [Chow PregEx Control; −30.6%, *p *= 0.010; 95% CI (−554.8, −51.89)] or a high‐fat diet [High‐fat Sedentary Control; −29%, *p *= 0.016; 95% CI (−541.3, −38.39)] alone (Figure 3c) compared with Chow Sedentary Control males. Although maternal growth restriction alone or a single maternal lifestyle factor reduced nephron endowment by 25%–30%, maternal lifestyle factors in growth‐restricted dams (≥1) and multiple maternal lifestyle factors (≥2) in normal birth weight dams reduced the number of nephrons by ≤45% (Figure 3c) compared with Chow Sedentary Control males; Chow Exercise Restricted [−43.4%, *p *< 0.0001; 95% CI (−681.6, −178.7)], High‐fat Sedentary Restricted [−44.7%, *p *< 0.0001; 95% CI (−694.3, −191.4)] and all High‐fat PregEx groups [−39%; 95% CI for Control (−632.4, −129.6) *p *= 0.001 and Restricted (−643.1, −140.2) *p *= 0.000]. Exercise in High‐fat dams did not alter the number of nephrons, irrespective of maternal birth weight, compared with Chow Sedentary Control males.

### 3.3 Branching morphogenesis genes

**FIGURE 4:**
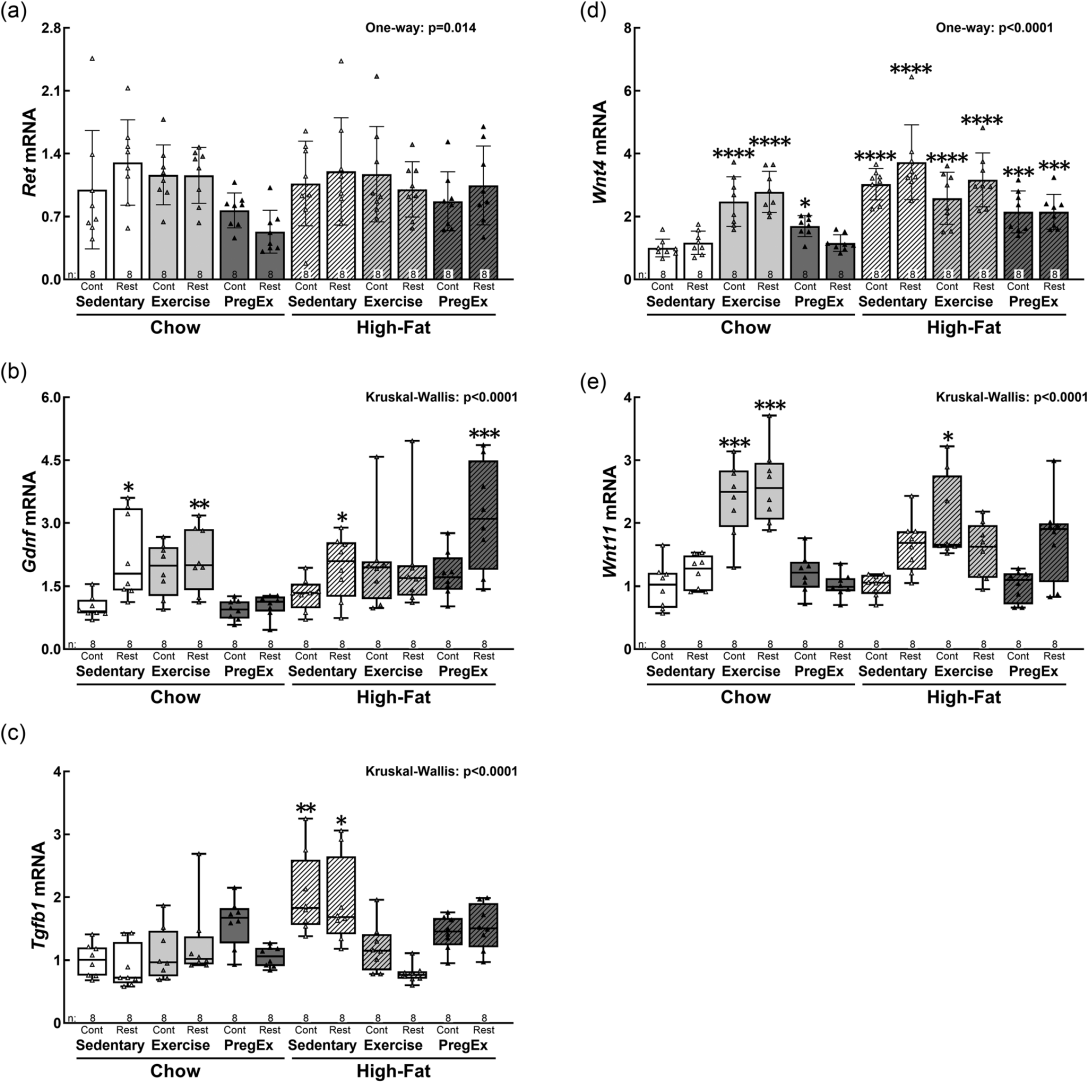
Abundance of renal branching morphogenesis markers. F2 male fetal kidney abundance of *Ret* (a), *Gdnf* (b), *Tgfb1* (c), *Wnt4* (d) and *Wnt11* (e) mRNA (*n* = 8 per group, with *n* = 1 representing one pup from one litter), whose mothers were Control (Cont) or Restricted (Rest). Data were analysed with a one‐way ANOVA with Dunnett's *post hoc* test (normally distributed) or the Kruskal–Wallis test with Dunn's *post hoc* test (not normally distributed) to identify differences between experimental groups compared with the control (Chow Sedentary Control). Data are presented as the mean ± SD or median (IQR). Significant differences between Chow Sedentary Control and experimental groups are indicated as follows: *p < 0.05, **p < 0.01, ***p < 0.001 and ****p < 0.0001. Detailed information [comparison group sample sizes and means, mean difference, 95% confidence interval and *p*‐values (where appropriate)] regarding significant differences can be found in the Statistical Summary Document.

Despite a statistically significant effect on *Ret* gene abundance (*p *= 0.014), no differences were detected by *post hoc* analysis between experimental groups and Chow Sedentary Control males (Figure 4a). *Gdnf* was not different in Control groups but was increased in all Restricted groups apart from Chow PregEx Restricted and High‐fat Exercise Restricted (Figure 4b): Chow Sedentary Restricted (+122%, *p *= 0.015), Chow Exercise Restricted (+115%, *p *= 0.010), High‐fat Sedentary Restricted (+96%, *p *= 0.048) and High‐fat PregEx Restricted (+217%, *p *= 0.000). *Tgfb1* was increased in High‐fat Sedentary Control (+104%, *p *= 0.005) and High‐fat Sedentary Restricted (+92%, *p *= 0.022) fetuses (Figure 4c). *Wnt4* was increased in Chow Exercise Control [+148%, *p *< 0.0001; 95% CI (0.28, 0.85)], Chow Exercise Restricted [+178%, *p *< 0.0001; 95% CI (0.38, 0.95)] and Chow PregEx Control [+70%, *p *= 0.028; 95% CI (0.02, 0.59)] males and in all High‐fat groups (Figure 4d): High‐fat Sedentary Control [+200%, *p *< 0.0001; 95% CI (0.46, 1.03)], High‐fat Sedentary Restricted (+272%, *p *< 0.0001; 95% CI (0.64, 1.20)], High‐fat Exercise Control [+158%, *p *< 0.0001; 95% CI (0.31, 0.88)], High‐fat Exercise Restricted [+217%, *P *< 0.0001; 95% CI (0.49, 1.06)], High‐fat PregEx Control [+115%, *p *= 0.000; 95% CI (0.17, 0.74)] and High‐fat PregEx Restricted [+115%, *p *= 0.000; 95% CI (0.18, 0.75)]. *Wnt11* was increased in Chow Exercise Control (+139%, *p *= 0.001), Chow Exercise Restricted (+160%, *p *= 0.000) and High‐fat Exercise Control (+107%, *p *= 0.012) fetuses (Figure 4e).

### 3.4 Angiogenic genes

**FIGURE 5:**
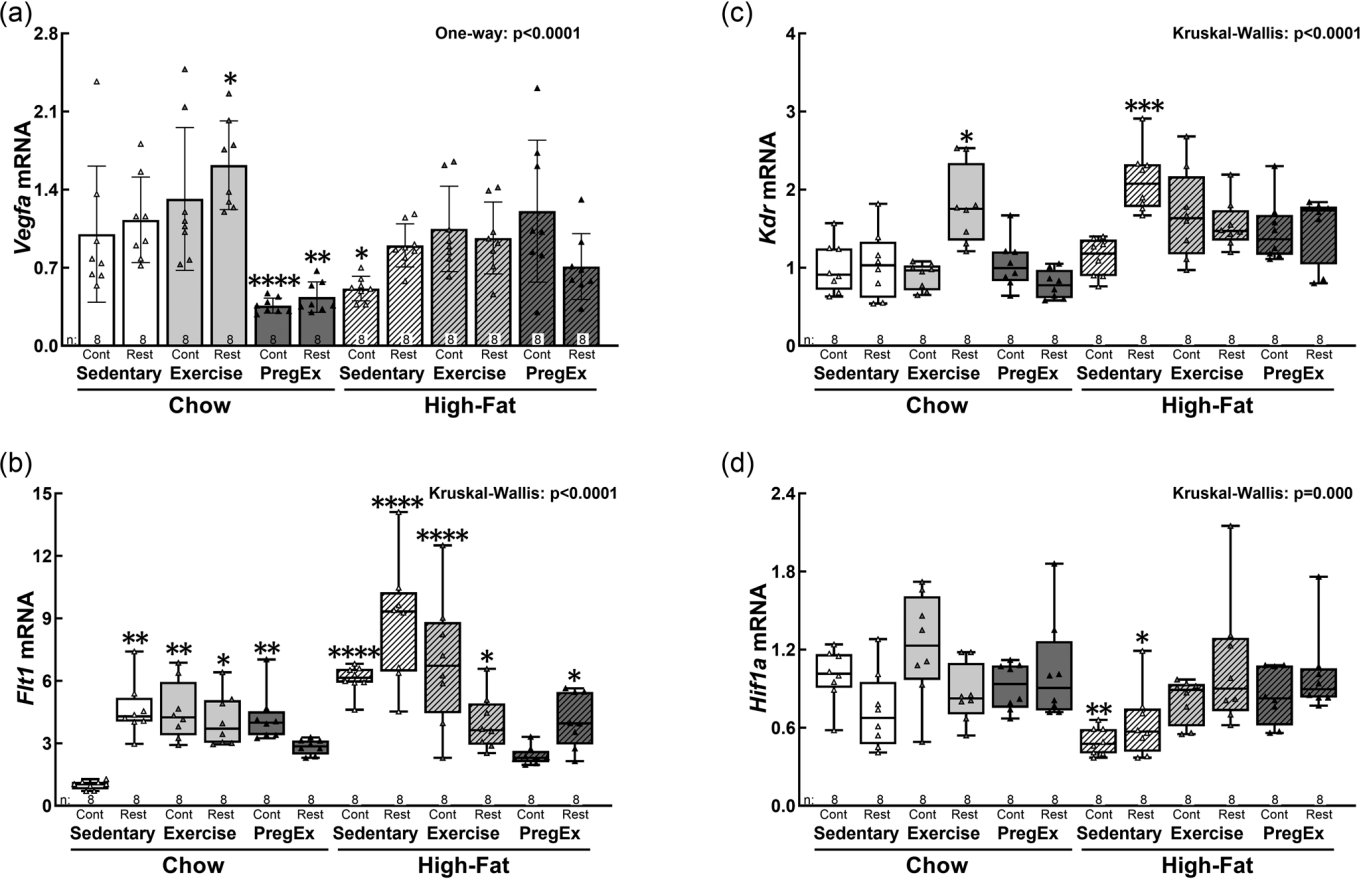
Abundance of renal angiogenesis markers. F2 male fetal kidney abundance of *Vegfa* (a), *Flt1* (b), *Kdr* (c) and *Hif1a* (d) mRNA (*n* = 8 per group, with *n* = 1 representing one pup from one litter), whose mothers were Control (Cont) or Restricted (Rest). Data were analysed with a one‐way ANOVA with Dunnett's *post hoc* test (normally distributed) or the Kruskal–Wallis test with Dunn's *post hoc* test (not normally distributed) to identify differences between experimental groups compared with control (Chow Sedentary Control). Data presented as the mean ± SD or median (IQR). Significant differences between Chow Sedentary Control and experimental groups are indicated as follows: *p < 0.05, **p < 0.01, ***p < 0.001 and ****p < 0.0001. Detailed information [comparison group sample sizes and means, mean difference, 95% confidence interval and *p*‐values (where appropriate)] regarding significant differences can be found in the Statistical Summary Document.

*Vegfa* was increased in Chow Exercise Restricted fetuses [+62%, *p *= 0.019; 95% CI (0.03, 0.48)] and reduced in Chow PregEx Control [−64%, *p *< 0.0001; 95% CI (−0.62, −0.17)], Chow PregEx Restricted [−57%, *p *= 0.001; 95% CI (−0.55, −0.10)] and High‐fat Sedentary Control [−49%, *p *= 0.025; 95% CI (−0.47, −0.02)] fetuses (Figure 5a). In contrast, *Flt1* was increased in all groups apart from Chow PregEx Restricted and High‐fat PregEx Control (Figure 5b): Chow Sedentary Restricted (+363%, *p *= 0.002), Chow Exercise Control (+355%, *p *= 0.003), Chow Exercise Restricted (+311%, *p *= 0.021), Chow PregEx Control (+323%, *p *= 0.009), High‐fat Sedentary Control (+509%, *p *< 0.0001), High‐fat Sedentary Restricted (+780%, *p *< 0.0001), High‐fat Exercise Control (+592%, *p *< 0.0001), High‐fat Exercise Restricted (+298%, *p *= 0.037) and High‐fat PregEx Restricted (+311%, *p *= 0.026). The other VEGF receptor, *Kdr*, was increased in Chow Exercise Restricted (+79%, *p *= 0.026) and High‐fat Sedentary Restricted (+112%, *p *= 0.001) fetuses (Figure 5c). In contrast, *Hif1a* was reduced in High‐fat Sedentary Control (−50%, *p *= 0.002) and High‐fat Sedentary Restricted (−37%, *p *= 0.049) fetuses (Figure 5d).

### 3.5 Proliferation and apoptosis genes

**FIGURE 6:**
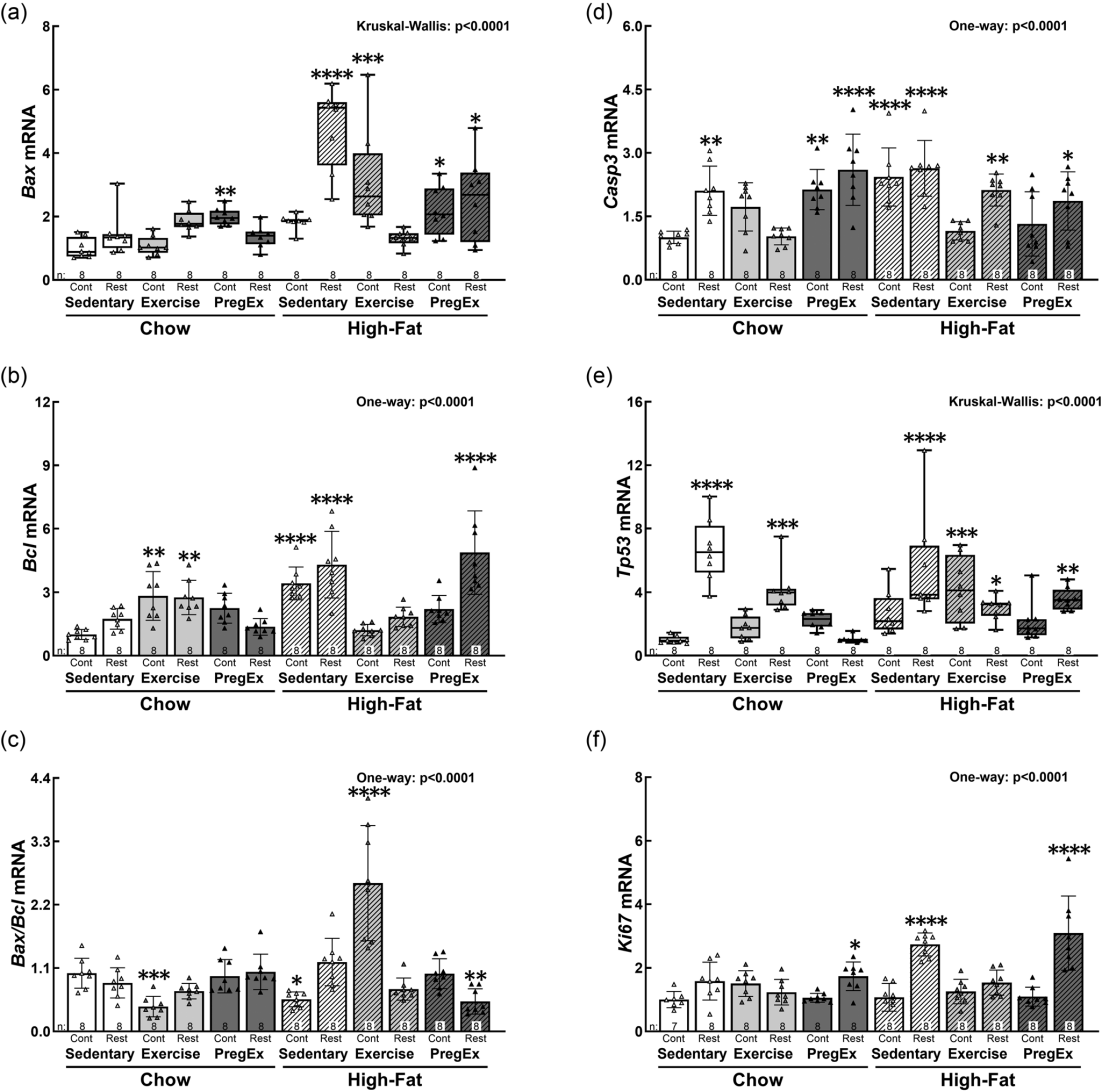
Abundance of renal apoptotic markers. F2 male fetal kidney abundance of *Bax* (a), *Bcl* (b), *Bax/Bcl* (c), *Casp3* (d), *Tp53* (e) and *Ki67* (f) mRNA (*n* = 8 per group for all data, except for *Ki67* abundance in Chow Sedentary Control where *n* = 7, with *n* = 1 representing one pup from one litter), whose mothers were Control (Cont) or Restricted (Rest). Data were analysed with a one‐way ANOVA with Dunnett's *post hoc* test (normally distributed) or the Kruskal–Wallis test with Dunn's *post hoc* test (not normally distributed) to identify differences between experimental groups compared with the control (Chow Sedentary Control). Data are presented as the mean ± SD or median (IQR). Significant differences between Chow Sedentary Control and experimental groups are indicated as follows: *p < 0.05, **p < 0.01, ***p < 0.001 and ****p < 0.0001. Detailed information [comparison group sample sizes and means, mean difference, 95% confidence interval and *p*‐values (where appropriate)] regarding significant differences can be found in the Statistical Summary Document.

The pro‐apoptotic gene *Bax* was increased in Chow PregEx Control (+100%, *p* = 0.009) and in all High‐fat groups except High‐fat Sedentary Control and High‐fat Exercise Restricted (Figure 6a): High‐fat Sedentary Restricted (+382%, *p *< 0.0001), High‐fat Exercise Control (+211%, *p* = 0.000), High‐fat PregEx Control (+120%, *p* = 0.012) and High‐fat PregEx Restricted (+154%, *p* = 0.011). In contrast, the anti‐apoptotic gene *Bcl* was increased in Chow Exercise Control [+183%, *p* = 0.002; 95% CI (0.51, 3.15)], Chow Exercise Restricted [+176%, *p* = 0.003; 95% CI (0.44, 3.08)], High‐fat Sedentary Control [+242%, *p *< 0.0001; 95% CI (1.10, 3.74)], High‐fat Sedentary Restricted [+330%, *p *< 0.0001; 95% CI (1.98, 4.62)], and High‐fat PregEx Restricted [+387%, *p *< 0.0001; 95% CI (2.55, 5.19)] fetuses (Figure 6b). However, the *Bax*/*Bcl* ratio, a marker of cellular apoptosis, was altered only in Chow Exercise Control [−57%, *p* = 0.000; 95% CI (−0.57, −0.13)], High‐fat Sedentary Control [−44%, *p* = 0.014; 95% CI (−0.48, −0.04)], High‐fat Exercise Control [+157%, *p *< 0.0001; 95% CI (0.36, 0.80)] and High‐fat PregEx Restricted [−48%, *p* = 0.004; 95% CI (−0.51, −0.07)] fetuses (Figure 6c). *Casp3* was increased in Chow Sedentary Restricted [+110%, *p* = 0.002; 95% CI (0.31, 1.89)], Chow PregEx Control [+113%, *p* = 0.001; 95% CI (0.34, 1.92)], Chow PregEx Restricted [+160%, *p *< 0.0001; 95% CI (0.81, 2.39)], High‐fat Sedentary Control [+143%, *p *< 0.0001; 95% CI (0.64, 2.22)], High‐fat Sedentary Restricted [+164%, *P *< 0.0001; 95% CI (0.84, 2.42)], High‐fat Restricted Exercise [+112%, *p* = 0.002; 95% CI (0.33, 1.91)] and High‐fat PregEx [+86%, *p* = 0.026; 95% CI (0.07, 1.65)] fetuses (Figure 6d). *Tp53* was increased in Chow Sedentary Restricted (+566%, *p *< 0.0001), Chow Exercise Restricted (+315%, *p* = 0.000) and all High‐fat groups except High‐fat Sedentary Control and High‐fat PregEx Control (Figure 6e): High‐fat Sedentary Restricted (+445%, *p *< 0.0001), High‐fat Exercise Control (+318%, *p* = 0.001), High‐fat Exercise Restricted (+200%, *p* = 0.018) and High‐fat PregEx Restricted (+257%, *p* = 0.001). *Ki67*, a marker of proliferation, was only increased in Chow PregEx Restricted [+73%, *p* = 0.049; 95% CI (0.00, 1.47)] and High‐fat PregEx Restricted [+209%, *p *< 0.0001; 95% CI (1.36, 2.82)] groups and in High‐fat Sedentary Restricted [+174%, *p *< 0.0001; 95% CI (1.01, 2.47)] fetuses (Figure 6f).

### 3.6 Stress‐responsive genes

**FIGURE 7:**
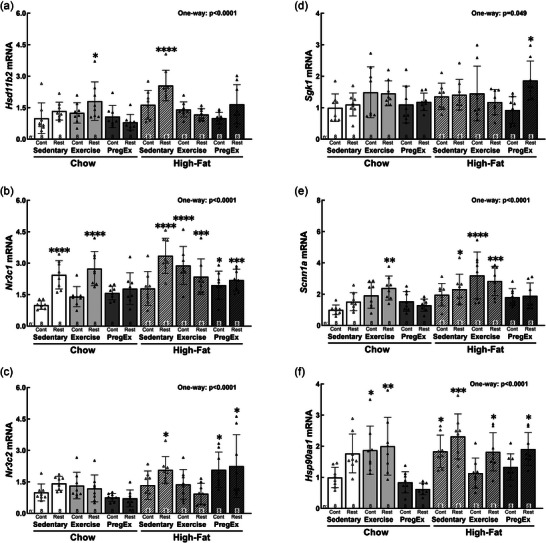
Abundance of renal stress markers. F2 male fetal kidney abundance of *Hsd11b2* (a), *Nr3c1* (b), *Nr3c2* (c), *Sgk1* (d), *Scnn1a* (e) and *Hsp90aa1* (f) mRNA (*n* = 8 per group, with *n* = 1 representing one pup from one litter), whose mothers were Control (Cont) or Restricted (Rest). Data were analysed with a one‐way ANOVA with Dunnett's *post hoc* test (normally distributed) to identify differences between experimental groups compared with the control (Chow Sedentary Control). Data are presented as the mean ± SD. Significant differences between Chow Sedentary Control and experimental groups are indicated as follows: *p < 0.05, **p < 0.01, ***p < 0.001 and ****p < 0.0001. Detailed information [comparison group sample sizes and means, mean difference, 95% confidence interval and *p*‐values (where appropriate)] regarding significant differences can be found in the Statistical Summary Document.

*Hsd11b2* abundance was increased in Chow Exercise Restricted [+81%, *p *= 0.030; 95% CI (0.03, 0.69)] and High‐fat Sedentary Control [+156%, *p *< 0.0001; 95% CI (0.30, 0.97)] fetuses (Figure 7a). The glucocorticoid receptor, *Nr3c1*, was increased in Sedentary Chow Restricted [+114%, *p *< 0.0001; 95% CI (0.23, 0.87)] and Exercise Chow Restricted [+174%, *p *< 0.0001; 95% CI (0.33, 0.97)] fetuses and in all High‐fat groups except High‐fat Sedentary Control (Figure 7b): High‐fat Sedentary Restricted [+234%, *p *< 0.0001; 95% CI (0.50, 1.14)], High‐fat Exercise Control [+190%, *p *< 0.0001; 95% CI (0.37, 1.01)], High‐fat Exercise Restricted [+136%, *p *= 0.000; 95% CI (0.20, 0.84)], High‐fat PregEx Control [+95%, *p *= 0.012; 95% CI (0.06, 0.70)] and High‐fat PregEx Restricted [+120%, *p *= 0.001; 95% CI (0.16, 0.80)]. In contrast, the mineralocorticoid receptor, *Nr3c2*, was increased only in High‐fat Sedentary Restricted [+107%, *p *= 0.016; 95% CI (0.06, 0.83)], High‐fat PregEx Control [+108%*, p *= 0.020; 95% CI (0.05, 0.82)] and High‐fat PregEx Restricted [+125%, *p *= 0.015; 95% CI (0.06, 0.83]) fetuses (Figure 7c). *Sgk1* abundance was increased only in High‐fat PregEx Restricted [+87%, *p *= 0.015; 95% CI (0.12, 1.62)] fetuses (Figure 7d). The α‐subunit of the epithelial sodium channel, *Scnn1a*, was increased in Chow Exercise Restricted [+139%, *p *= 0.008; 95% CI (0.27, 2.52)], High‐fat Sedentary Restricted [+132%, *p *= 0.014; 95% CI (0.19, 2.44)], High‐fat Exercise Control [+220%, *p *< 0.0001; 95% CI (1.07, 3.33)] and High‐fat Exercise Restricted [+184%, *p *= 0.000; 95% CI (0.71, 2.97)] fetuses (Figure 7e). *Hsp90aa1* abundance was increased in Chow Exercise Control [+87%, *p *= 0.027; 95% CI (0.07, 1.68)], Chow Exercise Restricted [+100%, *p *= 0.007; 95% CI (0.20, 1.81)], High‐fat Sedentary Control [+84%, *p *= 0.038; 95% CI (0.03, 1.65)], High‐fat Sedentary Restricted [+132%, *p *= 0.000; 95% CI (0.51, 2.13)], High‐fat Restricted Exercise [+82%, *p *= 0.044; 95% CI (0.01, 1.63)] and High‐fat PregEx Restricted [+91% *p *= 0.018; 95% CI (0.11, 1.72)] fetuses (Figure 7f).

### 3.7 Telomeres

**FIGURE 8:**
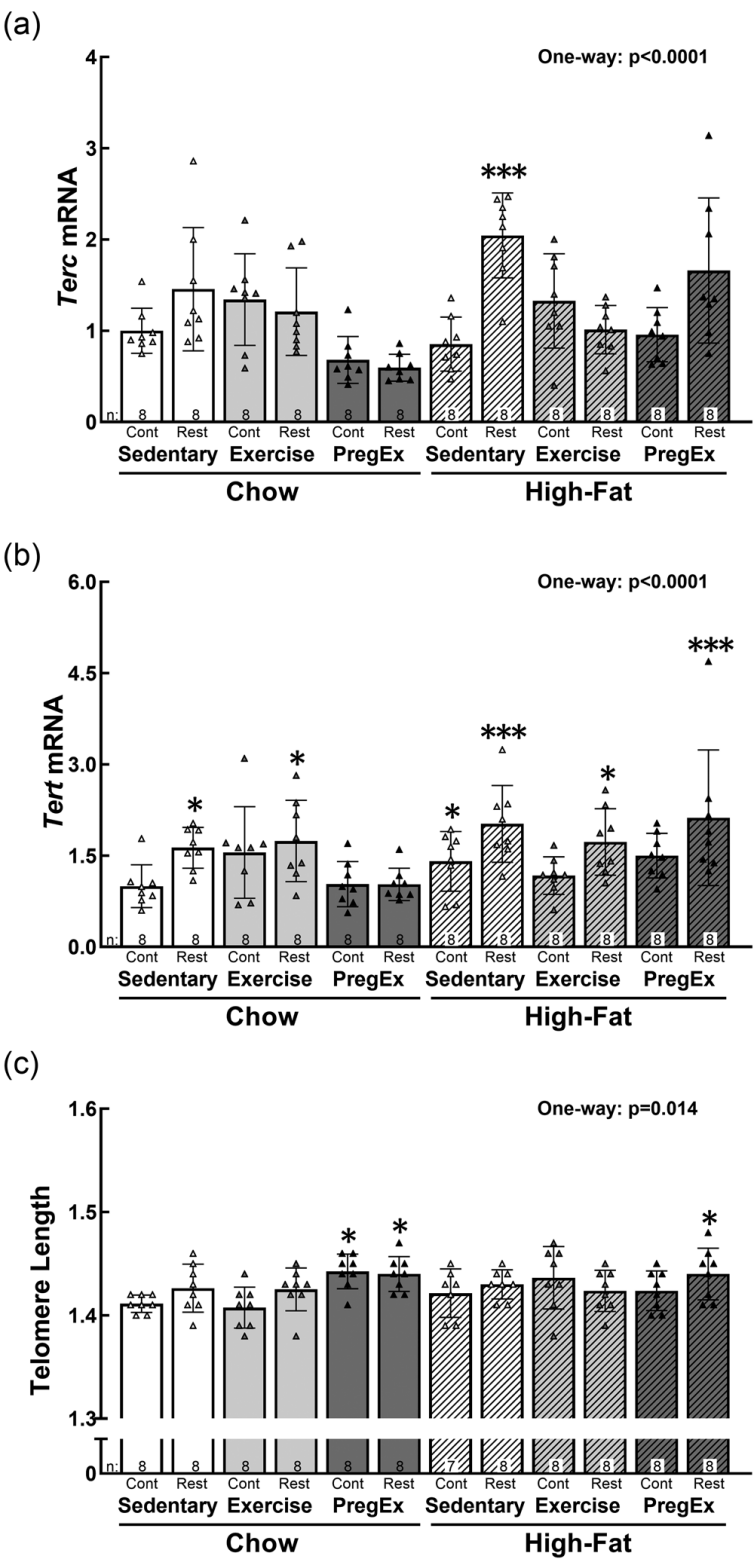
Abundance of renal telomere markers. Male fetal kidney abundance of *Terc* (a) and *Tert* (b) mRNA, in addition to telomere length (c) (*n* = 8 per group for all data except for telomere length in High‐fat Sedentary Control, where *n* = 7, with *n* = 1 representing one pup from one litter), whose mothers were Control (Cont) or Restricted (Rest). Data were analysed with a one‐way ANOVA with Dunnett's *post hoc* test (normally distributed) to identify differences between experimental groups compared with the control (Chow Sedentary Control). Data are presented as the mean ± SD. Significant differences between Chow Sedentary Control and experimental groups are indicated as follows: *p < 0.05, **p < 0.01, ***p < 0.001 and ****p < 0.0001. Detailed information [comparison group sample sizes and means, mean difference, 95% confidence interval and *p*‐values (where appropriate)] regarding significant differences can be found in the Statistical Summary Document.

One of the genes responsible for making telomerase, *Terc*, was increased in High‐fat Sedentary Restricted [+104%, *p *= 0.000; 95% CI (0.15, 0.70)] fetuses (Figure 8a). In contrast, the other gene, *Tert*, was increased in all Restricted groups apart from Chow PregEx Restricted fetuses and in High‐fat Sedentary Control [+41%, *p *= 0.022; 95% CI (0.03, 0.42)] (Figure 8b): Chow Sedentary Restricted [+63%, *p *= 0.022; 95% CI (0.02, 0.42)], Chow Exercise Restricted [+74%, *p *= 0.016; 95% CI (0.03, 0.43)], High‐fat Sedentary Restricted [+102%, *p *= 0.001; 95% CI (0.11, 0.51)], High‐fat Exercise Restricted [+73%, *p *= 0.013; 95% CI (0.04, 0.437)] and High‐fat PregEx Restricted [+112%, *p *= 0.001; 95% CI (0.11, 0.51)]. Telomere length was increased in all *PregEx* groups apart from High‐fat PregEx Control (Figure 8c): Chow PregEx Control [+44%, *p *= 0.026; 95% CI (0.00, 0.06)], Chow PregEx Restricted [+44%, *p *= 0.050; 95% CI (0.00, 0.06)] and High‐fat PregEx Restricted [+44%, *p *= 0.050; 95% CI (0.00, 0.58)].

### 3.8 Correlation analysis

**FIGURE 9:**
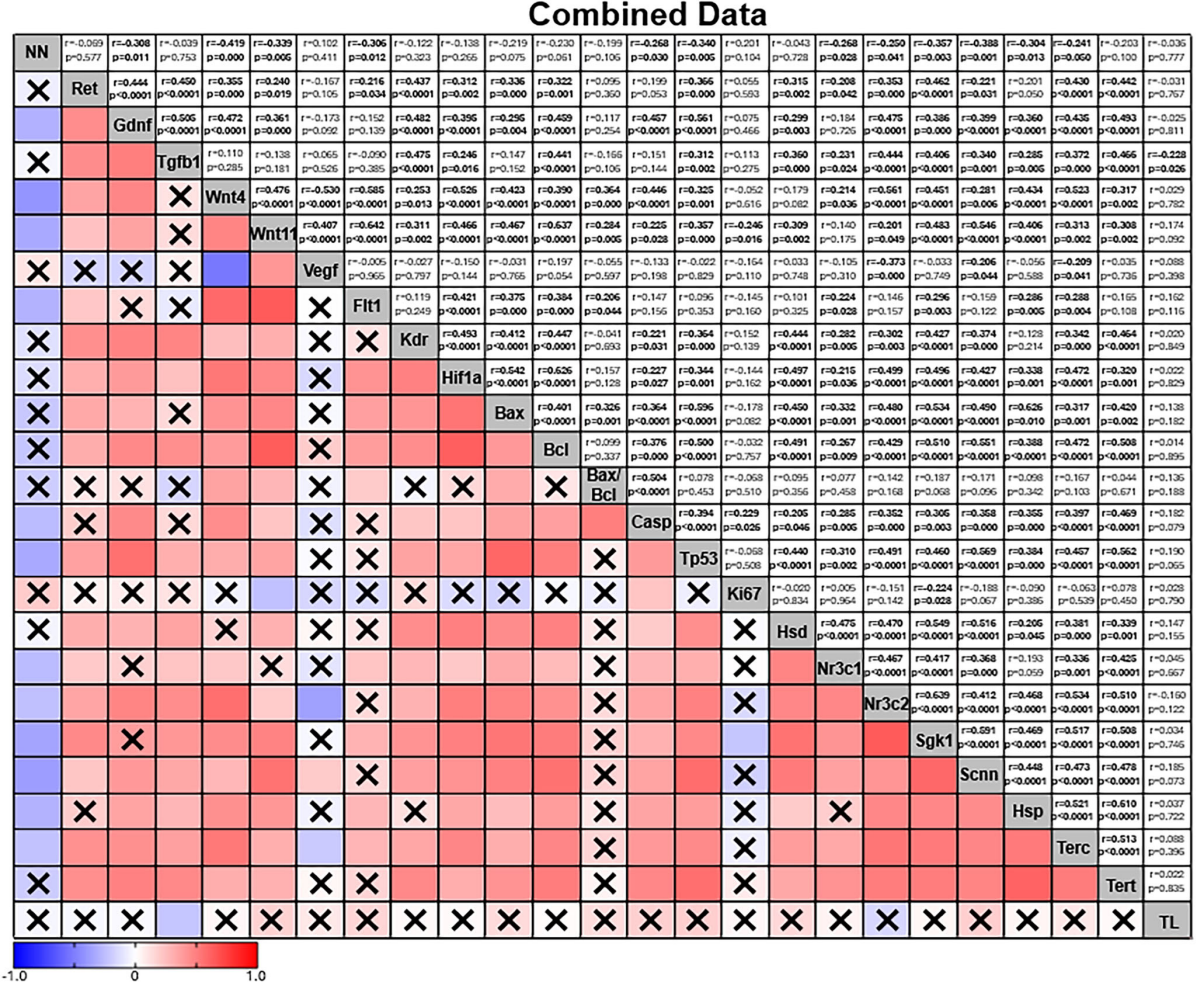
Correlation matrix in all groups combined between the number of nephrons and expression of genes, showing Spearman's non‐parametric correlation between the number of nephrons and the kidney genes explored in this study for all experimental groups combined. Spearman correlation coefficients (*r*; top number) and *p*‐value (bottom number) are displayed in the top squares. The colour of the bottom squares indicates the strength of the correlation (corresponding to the Spearman correlation coefficients). A cross through the bottom square indicates a non‐significant *p*‐value. Abbreviations: Casp, *Casp3*; Hsd, *Hsd11b2*; Hsp, *Hsp90aa1*; NN, number of nephrons; Scnn, *Scnna1*; TL, telomere length.

**FIGURE 10:**
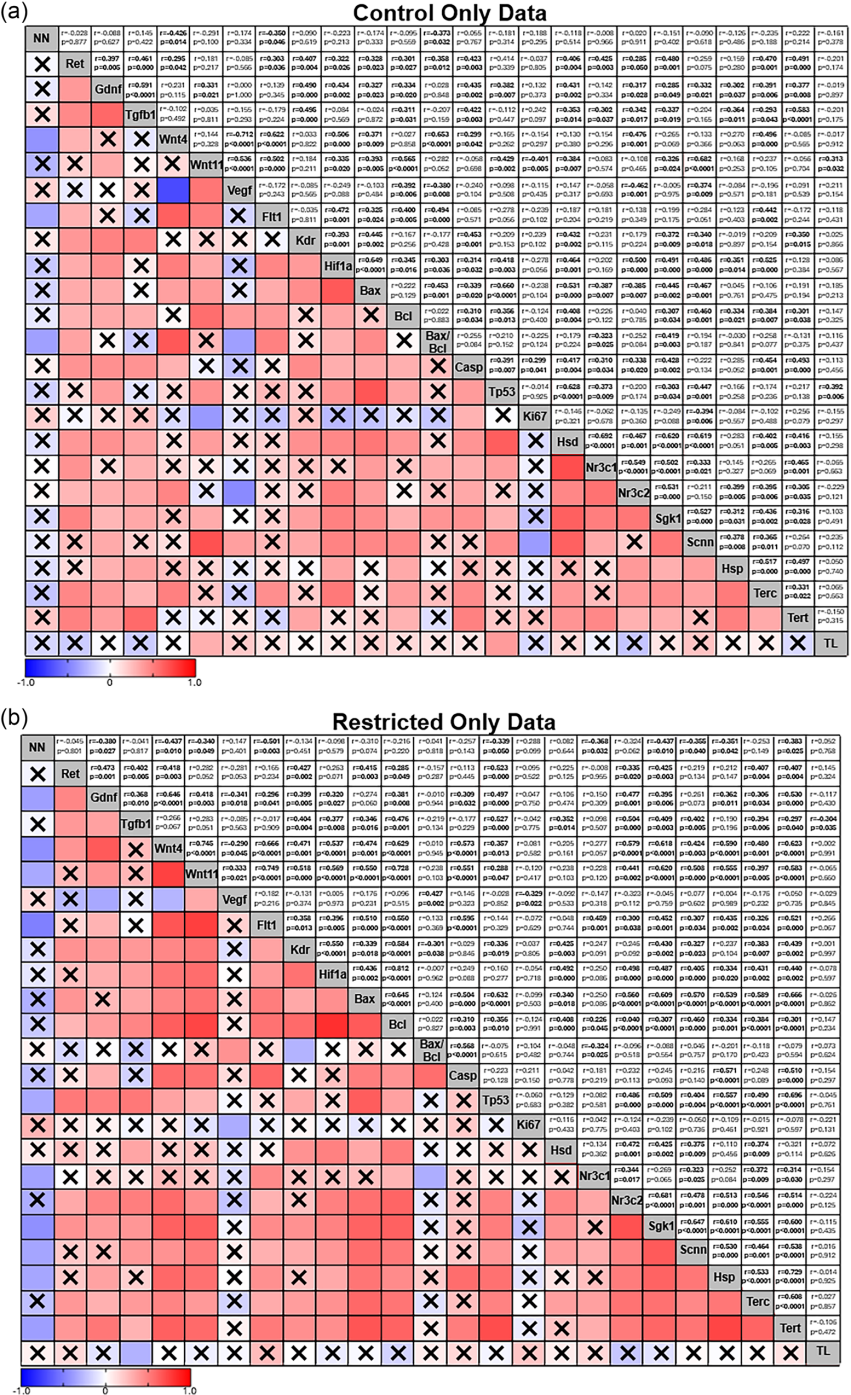
Correlation matrix between the number of nephrons and expression of genes in response to maternal birth weight, showing Spearman's non‐parametric correlation between the number of nephrons and the kidney genes explored in this study for Control (a) and Restricted (b) groups combining Diet and Exercise groups. Spearman correlation coefficients (*r*; top number) and *p*‐value (bottom number) are displayed in the top squares. The colour of the bottom squares indicates the strength of the correlation (corresponding to the Spearman correlation coefficients). A cross through the bottom square indicates a non‐significant *p*‐value. Abbreviations: Casp, *Casp3*; Hsd, *Hsd11b2*; Hsp, *Hsp90aa1*; NN, number of nephrons; Scnn, *Scnna1*; TL, telomere length.

**FIGURE 11:**
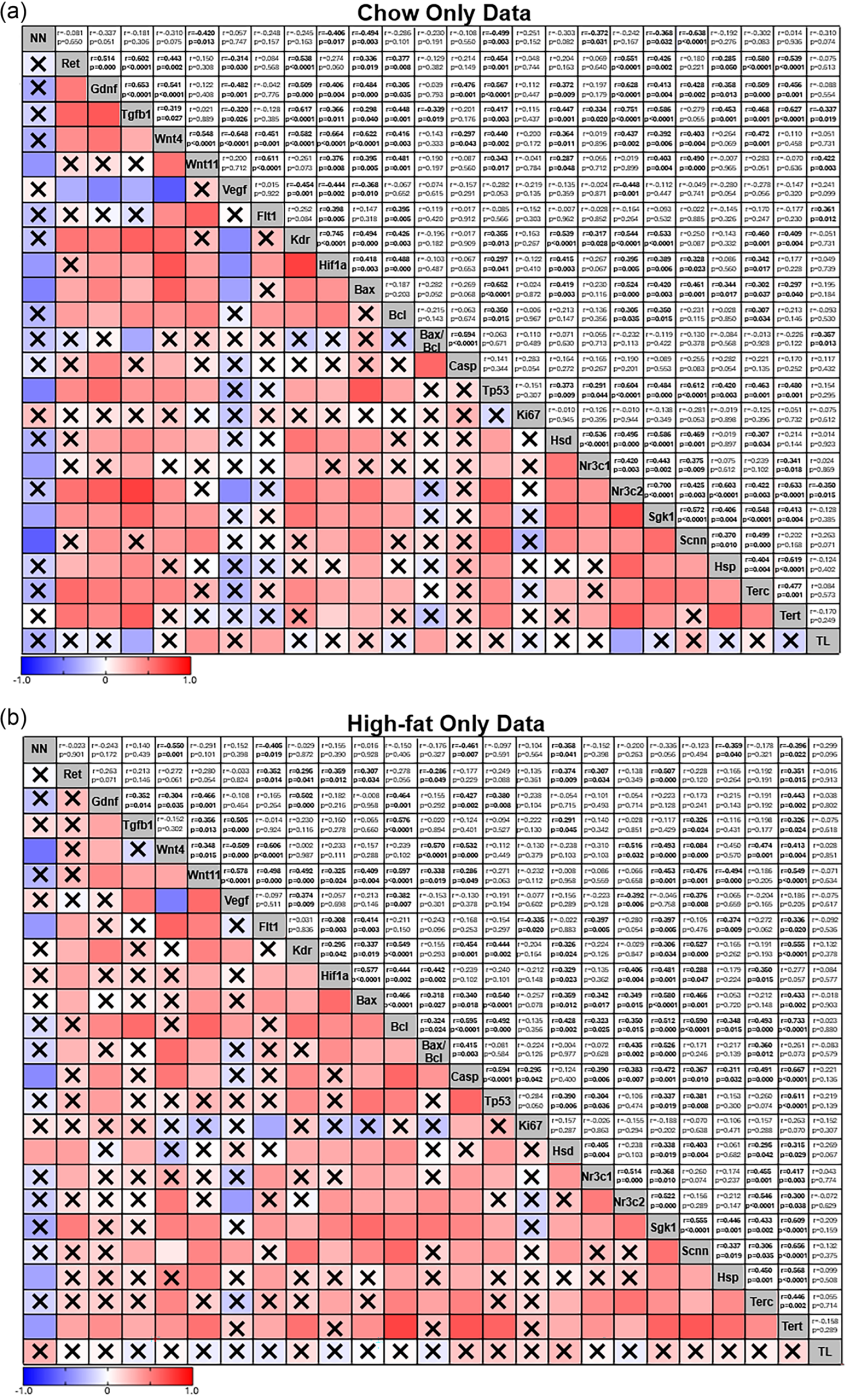
Correlation matrix between the number of nephrons and expression of genes in response to maternal diet, showing Spearman's non‐parametric correlation between the number of nephrons and the kidney genes explored in this study for Chow (a) and High‐fat (b) groups combining maternal birth weight and Exercise groups. Spearman correlation coefficients (*r*; top number) and *p*‐value (bottom number) are displayed in the top squares. The colour of the bottom squares indicates the strength of the correlation (corresponding to the Spearman correlation coefficients). A cross through the bottom square indicates a non‐significant *p*‐value. Abbreviations: Casp, *Casp3*; Hsd,  *Hsd11b2*; Hsp, *Hsp90aa1*; NN, number of nephrons; Scnn, Scnna1; TL, telomere length.

**FIGURE 12:**
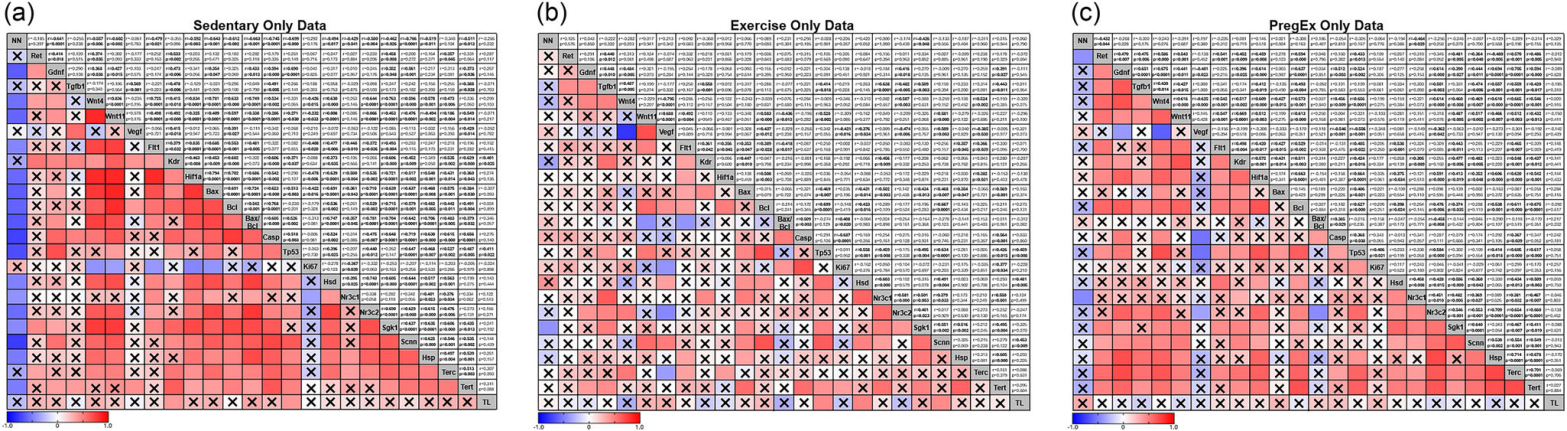
Correlation matrix between the number of nephrons and expression of genes in response to maternal exercise, showing Spearman's non‐parametric correlation between the number of nephrons and the kidney genes explored in this study for Sedentary (a), Exercise (b) and PregEx (c) groups combining maternal birth weight and Diet groups. Spearman correlation coefficients (*r*; top number) and *p*‐value (bottom number) are displayed in the top squares. The colour of the bottom squares indicates the strength of the correlation (corresponding to the Spearman correlation coefficients). A cross through the bottom square indicates a non‐significant *p*‐value. Abbreviations: Casp, *Casp3*; Hsd,  *Hsd11b2*; Hsp, *Hsp90aa1*; NN, number of nephrons; Scnn, *Scnna1*; TL, telomere length.

To explore the relationship between nephron number and the various genes examined in this study, we performed correlation analysis. Correlation analysis identified a weak‐to‐strong negative correlation between nephron endowment and several known genes that regulate nephrogenesis (Figure 9): *Gdnf* [*r* = −0.308, moderate, *p *= 0.011; 95% CI (−0.516, −0.07)], *Wnt4* [*r* = −0.419, moderate, *p *= 0.000; 95% CI (−0.604, strong, −0.192)], *Wnt11* [*r* = −0.339, moderate, *p *= 0.005; 95% CI (−0.541, −0.100)], *Flt1* [*r* = −0.306, moderate, *p *= 0.012; 95% CI (−0.514, −0.063)], *Casp3* [*r* = −0.268, weak, *p *= 0.030; 95% CI (−0.4843, −0.020)] and *Tp53* [*r* = −0.340, moderate, *p *= 0.005; 95% CI (−0.542, −0.101)]. Interestingly, the abundance of all stress‐responsive genes and *Terc* [*r* = −0.241, weak, *p *= 0.050; 95% CI (−0.460, 0.006)] have a weak‐to‐moderate negative correlation with nephron endowment: *Nr3c1* [*r* = −0.268, weak, *p *= 0.028; 95% CI (−0.4833, −0.023)], *Nr3c2* [*r* = −0.250, weak, *p *= 0.041; 95% CI (−0.468, −0.003)], *Sgk1* [*r* = −0.357, moderate, *p *= 0.003; 95% CI (−0.555, −0.121)], *Scnn1a* [*r* = −0.388, moderate, *p *= 0.001; 95% CI (−0.579, −0.156)] and *Hsp90aa1* [*r* = −0.304, moderate, *p *= 0.013; 95% CI (−0.512, −0.061)]. Group analysis seperating data based on maternal birth weight (Figure 10), diet (Figure 11), and exercise (Figure 12) highlight that these changes were largely associated with the correlations observed in the Restricted only (Figure 10b) and Sedentary only (Figure 12a) subgroups, because the other subgroup analyses [i.e. Control only (Figure 10a), Chow only (Figure 11a), High‐fat only (Figure 11b), Exercise only (Figure 12b) and PregEx only (Figure 12b)] report only very few significant correlations between genes examined and nephron endowment.

Interestingly, the Sedentary only groups (Figure 12a) had robust moderate‐to‐very strong correlations between many genes, whereas Exercise (Figure 12b) and PregEx (Figure 12c) had fewer significant correlations, with Exercise overall having fewer significant correlations than any other group.

## 4 DISCUSSIONlit

Nephron deficits occur following multiple maternal perturbations (Abdel‐Hakeem et al., 2008; Cuffe et al., 2018; Gray et al., 2010; Shen et al., 2010; Singh, Cullen‐McEwen, et al., 2007; Singh, Moritz, et al., 2007; Welham et al., 2002), including fetal growth restriction (Luyckx et al., 2013; Moritz et al., 2008; Wlodek et al., 2008), which places these individuals at an increased risk of developing hypertension and chronic kidney disease in later life. However, very few studies have explored the impact these perturbations have on the multi‐ and/or transgenerational programming of kidney development. In addition, whether adverse maternal lifestyle factors (i.e. high‐fat diet) can exacerbate this outcome or whether beneficial maternal lifestyle interventions (i.e. exercise) can prevent this transgenerational programming is unknown.

This study demonstrated that maternal growth restriction alone or a single maternal lifestyle factor in normal birth weight females (i.e. high‐fat feeding or exercise only during pregnancy) result in a nephron deficit of ∼30% in F2 male offspring, which is consistent with several animal models of developmental programming (O'Sullivan et al., 2013; Vehaskari & Woods, 2005; Wintour et al., 2003; Wlodek et al., 2007). We also demonstrated that maternal lifestyle factors in growth‐restricted dams, in addition to two or more maternal lifestyle factors in normal birth weight dams, reduce nephron endowment by ≤45%, which could have significant impacts on cardiorenal health if there is no increased maturation and/or growth prior to the cessation of nephrogenesis (∼PN10 in rats). Importantly, we demonstrated the benefits of exercise initiated prior to and continued throughout pregnancy on nephron endowment in dams fed a high‐fat diet, irrespective of maternal birth weight, which might highlight treatment strategies to maintain normal kidney growth and development in women at risk.

### 4.1 Impact of maternal growth restriction alone or a single maternal lifestyle factor on the number of fetal nephrons

Consistent with several animal models of developmental programming, maternal growth restriction alone [i.e. being born growth restricted (Chow Sedentary Restricted)] or a single maternal lifestyle factor in normal birth weight dams [i.e. consumption of a high‐fat diet in normal birth weight dams (High‐fat Sedentary Control), exercise prior to and during pregnancy in normal birth weight dams (Chow Exercise Control) or exercise only during pregnancy in normal birth weight dams (Chow PregEx Control)] reduced male F2 fetal nephron endowment by 25%–30%, which has been linked previously to adult‐onset cardiorenal disease (Luyckx et al., 2011).

The molecular mechanisms underpinning the nephron deficit in the present study appear to be dependent on whether the dam was growth restricted or whether the maternal lifestyle factor in normal birth weight dams was beneficial (i.e. exercise) or detrimental (i.e. high‐fat feeding) to maternal health. Specifically, in fetuses of growth‐restricted chow‐fed dams [Chow Sedentary Restricted] and normal birth weight high‐fat fed dams [High‐fat Sedentary Control], the kidney appears to be immature and undergoing significant remodelling on E20, as evident by increased markers of branching morphogenesis (*Gdnf* in Chow Sedentary Restricted) and kidney development (*Wnt4* in High‐fat Sedentary Control and Chow PregEx Control; *Tgfb1* in High‐fat Sedentary Control). Additionally, reduced *Vegfa* abundance (Chow PregEx Control and High‐fat Sedentary Control) and increased VEGF receptor (*Flt1*) abundance (Chow Sedentary Restricted, Chow PregEx Control and High‐fat Sedentary Control) suggest compromised and/or delayed renal angiogenesis. Increased abundance of genes that regulate apoptosis, without increases in proliferation markers, further suggests compromised kidney development: increased *Casp3* (Chow Sedentary Restricted and High‐fat Sedentary Control) and increased *Tp53* (Chow Sedentary Restricted). However, it is not known whether these changes in genes result in a permanent reduction in nephron endowment, which requires further investigation at a time point when nephrogenesis has ceased (i.e. PN10), especially given that the reduced *Bax/Bcl* ratio in High‐fat Sedentary Control males might mitigate some of the deleterious effects of increases is apoptotic genes. Nevertheless, these findings are consistent with our previous reports in first‐generation growth‐restricted offspring (Cuffe et al., 2018) highlighting that these changes are likely to be programmed epigenetically (Doan et al., 2021, 2024) and are transmitted to the F2 offspring, potentially via the placenta (Mangwiro, Briffa et al., 2018, 2021; Mangwiro, Cuffe et al., 2018, 2019), which requires further investigation using ChIPseq (histone modifications), proteomics (post‐translational modifications) and methylation assays.

Of note, our findings regarding the reduced fetal nephron endowment in high‐fat fed dams (i.e. High‐fat Sedentary Control) differ from the recent findings by Zhou et al. (2021), who report no effect on nephron endowment in high‐fat dams. This contrast in findings is likely to be attributable to the type of high‐fat diet used [60% (Zhou et al., 2021) vs. 43% (present study) digestible energy from lipids] and the method of estimating the number of nephrons [counting nephrons in five areas from three sections (Zhou et al., 2021) vs. the physical dissector/fractionator method (present study)].

Maternal exercise before and during pregnancy in normal birth weight chow‐fed dams [Chow Exercise Control], in contrast, results in gene changes that suggest an attempt to enhance nephrogenesis. Specifically, the kidney still appears to be undergoing development (increased *Wnt4*), ureteric branching morphogenesis (increased *Wnt11*) and endothelial differentiation (increased *Flt1*), which are likely to be an attempt to enhance nephron endowment (Eremina & Quaggin, 2004; Halt & Vainio, 2014). This is also highlighted by reduced markers of apoptosis (increased *Blc2*), suggesting that the kidney is still undergoing significant remodelling, potentially by inhibiting apoptosis to prolong cell proliferation and increase the number of cells (Ho, 2014). These changes, overall, might explain why the number of nephrons in Chow Exercise Control was not statistically different from Chow Sedentary Control fetuses, despite a 25% reduction in nephron endowment, because compensatory mechanisms are in place to enhance renal development.

### 4.2 Impact of maternal lifestyle factors in growth‐restricted dams and multiple lifestyle factors in normal birth weight dams on the number of fetal nephrons

Consistent with our hypothesis, maternal lifestyle factors in growth‐restricted dams, in addition to two or more maternal lifestyle factors in normal birth weight dams, had a more profound impact on nephron endowment, which was reduced by ≤45% and was often associated with more overt gene changes than fetuses of dams that were exposed to maternal growth restriction alone or to a single maternal lifestyle factor in normal birth weight dams. Specifically, maternal lifestyle factors in growth‐restricted dams [i.e. high‐fat diet (High‐fat Sedentary Restricted), exercise in chow‐fed dams (Chow Exercise Restricted) or exercise only during pregnancy in high‐fat dams (High‐fat PregEx Restricted)] resulted in the exacerbation of the gene changes detected in Chow Sedentary Restricted fetuses, which further suggests that the kidney is still undergoing profound remodelling. Gene changes suggest that kidney development is ongoing (increased *Wnt4*), as evidenced further by increased markers of branching morphogenesis (*Gdnf*), ureteric branching morphogenesis (*Wnt11*; Chow Exercise Restricted only), angiogenesis (*Vegf*; High‐fat Sedentary Restricted), endothelial differentiation (*Kdr* and *Flt1*) and renal apoptosis (*Trp53*, *Bax* and *Casp3*) that was not overcome by increased *Bcl2*, which coincides with increased proliferation (*Ki67*; High‐fat Sedentary Restricted and High‐fat PregEx Restricted). In addition to changes in known markers of nephrogenesis, we also report significant changes in numerous stress‐related genes following maternal lifestyle factors in growth‐restricted dams, which might have independent impacts on nephron endowment and program adult‐onset salt‐sensitive hypertension. However, these changes are independent of changes in fetal steroid concentrations (Mangwiro et al., 2021), and very little is known about the impact of these genes on kidney development, which requires further investigation. Interestingly *Tert* abundance, the rate‐limiting step in telomerase activation, was increased in all Restricted dams exposed to maternal lifestyle factors (apart from Chow PregEx Restricted), which suggests increased telomerase activity, hence telomere elongation. However, increased telomere length was observed only in Chow PregEx Restricted and High‐fat PregEx Restricted fetuses. The impact of this telomere elongation on nephron endowment is unknown, although it is possible that it might be beneficial to long‐term kidney function, because previous studies have demonstrated telomere shortening in chronic kidney disease (Park et al., 2021). Overall, these data highlight that maternal lifestyle factors (i.e. high‐fat diet and/or exercise) in growth‐restricted dams result in more severe alterations to fetal kidney development. However, whether these changes are sustained throughout nephrogenesis and/or program adult kidney dysfunction remains unknown and should be explored in future studies.

In contrast to the large gene changes observed with maternal lifestyle factors in Restricted dams described above, there were very few changes in normal birth weight dams that were exposed to two maternal lifestyle factors (i.e. High‐fat PregEx Control) despite a 38% reduction in nephron endowment. Many of the gene changes suggest that the kidney is still undergoing development, with increased markers of renal growth (*Wnt4*) and apoptosis (*Bax*). This highlights that other pathways might contribute to the reduced nephron endowment in these fetuses. For example, it is possible that maternal nutrient availability and/or fetal oxygen delivery (Wilkinson et al., 2015) would be reduced with the sudden onset of exercise during pregnancy at a time of rapid differentiation that is close to the onset of kidney development in the rat (E11; Seely, 2017), which could lead to the reallocation of oxygen and nutrients away from the developing foetus and redirect them to metabolically active maternal tissues; findings which are in agreement with recent publications highlighting that maternal exercise can compromise fetal development (Lovell et al., 2024; Sene et al., 2021; Son et al., 2019). However, given that hypoxia and nutrient transporter abundances were not characterized in the present study, this highlights the need for additional studies investigating nutrient transporter abundance and function, in addition to hypoxic markers, following PregEx, which might provide additional insights into how nephrogenesis is compromised, especially given that we have reported previously that PregEx dysregulates placental nutrient transporters (Mangwiro et al., 2019). In addition, how PregEx affects nephron endowment once nephrogenesis is completed postnatally needs to be explored.

### 4.3 Impact of exercise before and during pregnancy in overweight mothers

Notably, we demonstrated that maternal exercise prior to and throughout pregnancy only in high‐fat fed dams (i.e. High‐fat Exercise Control and High‐fat Exercise Restricted) did not alter fetal nephron endowment. Why this differs from females on a chow diet is not known. This normalization of nephron endowment coincides with gene markers that suggest improved renal remodelling: increased renal growth (*Wnt4*), enhanced ureteric branching morphogenesis (*Wnt11*; High‐fat Exercise Control), increased VEGF receptor (*Flt1*), highlighting that endothelial cell differentiation, capillary formation and tubular epithelial proliferation are ongoing, and increased apoptotic markers, highlighting that the kidney is undergoing remodelling (*Trp53*; *Casp3* in High‐fat Exercise Restricted; and *Bax* and *Bax/Bcl* in High‐fat Exercise Control). However, it is likely that these changes do not completely explain the underlying mechanisms behind the protective impact on nephron endowment, which might work in concert with other maternal factors, such as increased nutrient reserves and/or maternal environmental factors. This highlights that additional studies are required to determine the mechanism(s) that are responsible, which might provide targeted therapeutic options for individuals born prior to the completion of nephrogenesis (such as pre‐term birth).

## 5 CONCLUSION

This study has demonstrated that maternal lifestyle factors in normal birth weight (at least two factors) and growth‐restricted (at least one factor) dams compromise fetal nephron endowment to a greater extent than fetuses exposed to maternal growth restriction alone or only one maternal lifestyle factor in normal birth weight dams (38%–45% vs. 25%–31%, respectively). Most importantly, we demonstrate the protective benefits of maternal exercise on maintaining a normal nephron endowment, but only when initiated prior to pregnancy and continued throughout pregnancy if the mother consumes a high‐fat diet. The mechanism behind this is likely to involve a combination of enhanced known regulators of nephrogenic genes in concert with enhanced nutrient availability to promote normal kidney development. Understanding these underlying mechanism(s) might offer therapeutic interventions and guidance for infants born with a reduced number of nephrons (such as pre‐term and growth‐restricted infants) to improve their own and their children's long‐term cardiorenal health breaking the cycle of transgenerational programming.

## AUTHOR CONTRIBUTIONS

Conception and design: Mary E. Wlodek, Karen M. Moritz, Glenn D. Wadley, Jessica F. Briffa, James S.M. Cuffe and Fadi Charchar. Acquisition of data: Jessica F. Briffa, Sogand S. Gravina, Viktoria F. Richter, Dayana Mahizir, Kristina Anevska, Yeukai T.M. Mangwiro and Deanne H. Hryciw. Analysis and interpretation of data: all authors. Writing, review and/or revision of the manuscript: all authors. All authors approved the final version of the manuscript and agree to be accountable for all aspects of the work in ensuring that questions related to the accuracy or integrity of any part of the work are appropriately investigated and resolved. All persons designated as authors qualify for authorship, and all those who qualify for authorship are listed.

## CONFLICT OF INTEREST

None declared.

## Supporting information




**Table S1**. Birth weight raw data. F1 Control (Cont; 38 litters) and Restricted (Rest; 40 litters) average female litter body weight at birth. Data were analysed with an unpaired *t*‐test.

**Table S2**. Postnatal body weight raw data of Figure 2a. F1 Control (Cont) and Restricted (Rest) female postnatal (PN) body weights through to weaning (58 and 56 females, respectively). Data were analysed with a mixed‐effects two‐way ANOVA.

**Statistical Summary Document**. Document highlighting the statistical comparisons for all significant data, which includes the sample size and mean for each group for comparison, the mean difference, 95% confidence interval, and *p*‐value (where appropriate). Each figure is presented as a separate tab in the Excel spreadsheet.

## Data Availability

The data that support the findings of this study are available from the corresponding author upon reasonable request.
